# Optimizing hemostasis in HoLEP surgery: retrospective review of selective bipolar plasmakinetic technology guided by bladder irrigation fluid color

**DOI:** 10.1007/s00345-024-05130-x

**Published:** 2024-07-18

**Authors:** Hengda Hu, Wenpu Chen, Weixiong Ma, Chengshuai Yu, Qirui He, Jinrong Tang, Guofeng Yu

**Affiliations:** Department of Urology, Shanghai Jinshan District Central Hospital, Jinshan District Central Hospital affiliated to Shanghai University of Medicine & Health Sciences, Jinshan Branch of the Sixth People’s Hospital of Shanghai, 147 Jiankang Road, Jinshan District, Shanghai, China

**Keywords:** HoLEP, Bipolar plasmakinetic, Hemostasis

## Abstract

**Object:**

To evaluate the effectiveness of selective bipolar plasmakinetic technology based on bladder irrigation fluid color on hemostasis in HoLEP surgwery

**Methods:**

A total of 209 patients who underwent HoLEP surgery from October 2021 to July 2023 were included and divided into Hemostasis Management Group and control group. the color of the irrigation fluid was categorized into 5 levels and the bipolar plasmakinetic technology was applied when the color came to level 4 or up. The following was analyzed: postoperative use of balloon compression, blood loss, irrigation time, length of hospital stay, and the number of a second operation.

**Results:**

Only 4 patients in Hemostasis Management Group required postoperative urinary catheter balloon compression, while there are 15 in the control group(p=0.03). The average irrigation time for patients in the HM Group with bipolar plasmakinetic hemostasis was 21.88±13.76 hours, compared to that in patients with catheter balloon compression(p=0.007).

**Conclusion:**

Based on the bladder irrigation color chart, the selective application of bipolar plasmakinetic hemostasis led to a significant reduction in the number of patients requiring postoperative bladder catheter balloon compression. Secondly, the irrigation time of patients who underwent bipolar plasmakinetic hemostasis also decreased.

## Introduction

Holmium Laser Enucleation of the Prostate (HoLEP) has emerged as an innovative surgical technique and has garnered preference as one of the primary methods rather than Transurethral resection of the prostate (TURP)for addressing BPH [1, 2]. This preference stems from its distinctive advantages, including minimal surgical trauma, reduced intraoperative bleeding, and favorable therapeutic outcome [2–4].

However, intraoperative bleeding during HoLEP surgery still remains a persistent and potentially life-threatening complication, underscoring the critical clinical significance and research value of strategies to mitigate bleeding risk. Current research suggests that severe intraoperative bleeding in this surgery can be attributed to factors such as such as patients with larger prostates, receiving antithrombotic therapy, and the surgeon’s lack of skill [5, 6].

Intraoperative hemostasis involves the use of lasers or bipolar plasmakinetic technology. Laser is utilized for the excision of the prostate gland and hemostasis, and bipolar plasmakinetic is employed at the end of the surgery usually when laser does not provide effective hemostasis [7]. it’s essential to consider that the application of bipolar plasmakinetic significantly extends surgical time and increases surgical costs. Consequently, determining the necessity of bipolar plasmakinetic becomes a pivotal concern.

In our study, we propose a method to assess the necessity for bipolar plasmakinetic by monitoring the color of the bladder irrigation fluid during surgery. This method establishes an effective intraoperative hemostasis protocol, which has demonstrably reduced intraoperative bleeding and bladder irrigation time, minimized the occurrence of postoperative urethral catheter balloon compression.

## Methods and patient population

### Study population and study design

This study was approved by the Ethics Committee of the Jinshan Branch of the Sixth People’s Hospital of Shanghai. It included a total of 209 patients underwent HoLEP surgery at our hospital by a single surgeon with 40 cases of surgical experience beforehand from October 2021 to July 2023 retrospectively by reviewing patient files. A holmium laser (Lumenis Inc, Palo Alto, CA) with 500-µm laser fibre was applied in these operations. In the process of incising the gland, the laser is set to 90 W (3.0 J 30 Hz), while in hemostasis mode, it is set to 32 W (0.8 J 40 Hz).

Among these patients, those who underwent surgery strictly following the method described below (Intraoperative Hemostasis Management) during the period from December 2022 to May 2023 were defined as the Hemostasis Management Group (HM group), comprising 105 patients. Patients who underwent surgery before this defined period were considered the Control Group, comprising 108 patients. All patients underwent preoperative MRI or ultrasound examinations to measure prostate volume. Preoperatively, blood samples were collected to measure PSA levels and coagulation parameters. In cases where malignant prostate conditions were suspected, prostate biopsies were performed prior to HoLEP surgery to confirm benign pathology. 3-lobe technique was used in the surgery generally.

### The operation and immediate post-operation hemostasis management

**Fig. 1:**
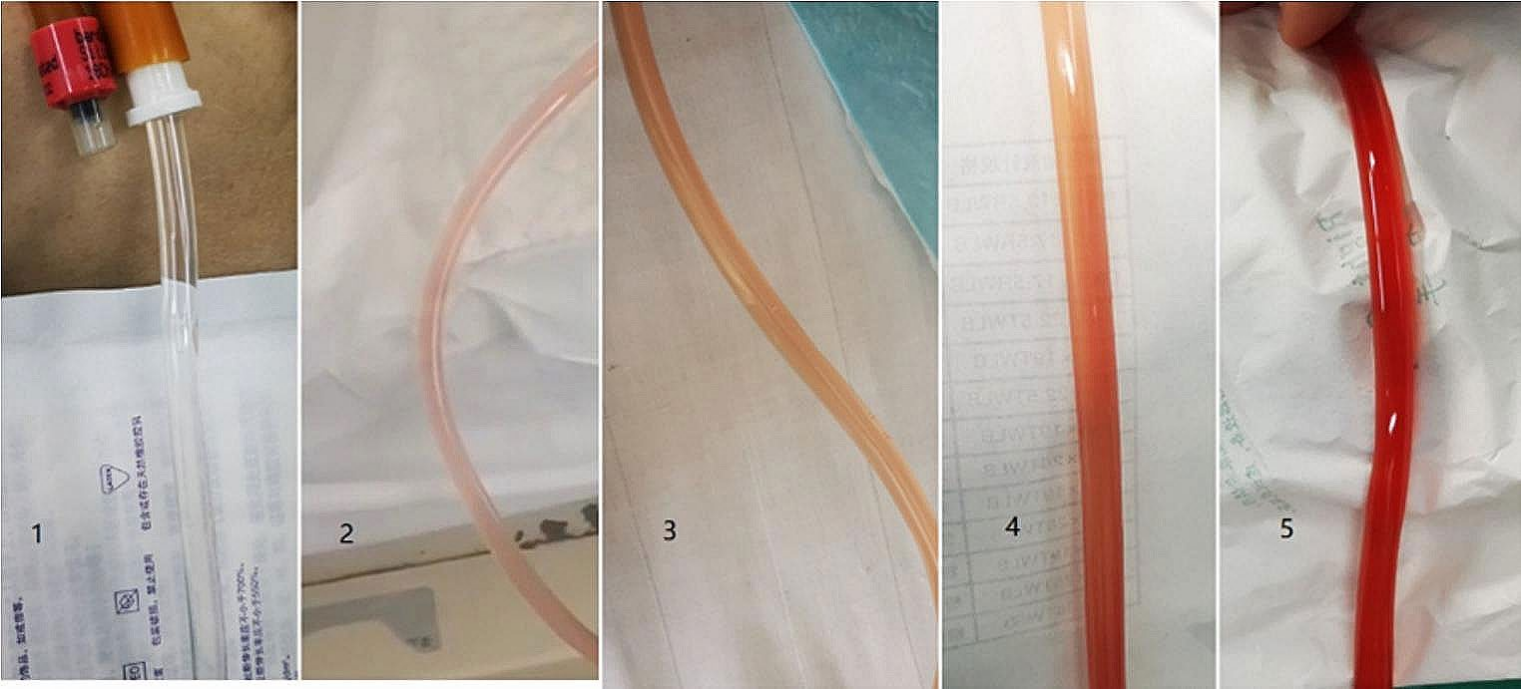
5 levels color of the irrigation chart

Bladder irrigation was performed using physiological saline at the end of surgery, and the color of the irrigation fluid was continuously observed for 2–3 min. The color observed during this period was noted. We categorized the color of the irrigation fluid into 5 levels, and when the color reached level 4 or higher(The patients’ blood pressure and heart rates should be controlled in the normal range at this time), indicating a severe bleeding and necessitated an immediate use of bipolar plasmakinetic (80 W, Olympus, Tokyo, Japan). Fig. 1.

The strategies for hemostasis are as follows: (1) Firstly, focus on the location where intraoperative hemostasis is inadequate. (2) If there are multiple bleeding points and it is difficult to determine their exact locations, hemostasis should be emphasized in the directions of 3–4 o’clock and 8–9 o’clock, as well as at the junction between the bladder and prostate. (3) Additionally, hemostasis should be conducted generally at the location of middle lobe. (4) Finally, assess the overall effectiveness of hemostasis by observing the color of bladder irrigation fluid once again.

### Post-operative management

Postoperatively, all patients, whether or not bipolar plasmakinetic was used, received routine bladder irrigation. Patients who received bladder compression would get traction with tape.

The nurses would inspect the ward every 4 h and stop the bladder irrigation if the color of the irrigation fluid is lighter than level 2. Generally, irrigation continued until the second day after surgery. If the irrigation fluid remained reddish in color, the bladder irrigation duration was extended as necessary. Those who received a balloon compression were treated with the same strategy to stop the balloon compression.

### Parameters

The primary objective of this study was to compare bleeding control outcomes in patients undergoing rigorous surgical hemostasis management.

Data collected included patient demographics (1) age, (2) preoperative PSA levels(ng/ml), (3) preoperative and postoperative hemoglobin levels(g/L), (4) preoperative measured prostate weight(pre-weight, g), (5) postoperative measured prostate weight(post-weight, g).(6) surgery time (min).

Bleeding control outcomes in patients were assessed through the following indicators: (1) postoperative use of balloon compression, (2) blood loss(g/L), (3) postoperative bladder irrigation duration (irrigation time, hour), (4) length of hospital stay(day), and (5) rate of secondary surgeries.

The formula for calculating preoperative prostate weight was as follows: width × height × length × π/6 [8]. Measurements of the three prostate dimensions were obtained from MR or ultrasound images of the patients. Blood loss was calculated by the difference between the hemoglobin level on the first day after surgery and the preoperative hemoglobin level.

### Statistical analysis

Data analysis was performed using GraphPad Prism 9 or SPSS 26.0 for Windows. Categorical data were examined using the Chi-square test and the continuous variable was assessed using the independent sample t-test. When comparing multiple groups of data, one-way ANOVA analysis was applied. *p* < 0.05 was considered to indicate a statistically significant difference, and the measurement data of normal distribution were represented by (mean ± SD).

## Results

1. This study included a total of 209 patients, with 105 patients in the HM group and 104 patients in the control group. The average age of patients in the hemostasis management group was 72.73 ± 7.90, while patients in the Control Group had an average age of 72.93 ± 7.58. There was no significant difference in age between the two groups (p value = 0.553). The PSA levels were 4.66 ± 4.11 in the HM Group and 5.59 ± 5.82 in the Control Group, with no statistical difference observed (*p* = 0.184). There was no statistical difference in pre-weight, surgery time, blood loss, post-weight, post-operative hospital day, and irrigation time and the detailed data were listed in Table 1. There was no transfusion in this research.
2. Among 105 patients in the HM Group, 8 patients had darker bladder irrigation fluid colors during surgery and receive a hemostasis with bipolar plasmakinetic technology. Only 4 patients in this group underwent postoperative urinary catheter balloon compression and none of them receive a hemostasis with bipolar plasmakinetic technology beforehand. In the Control Group, 15 patients received postoperative urinary catheter balloon compression. The difference in the rate of postoperative balloon compression between the two groups was statistically significant (*p* = 0.03, Table 1). There were no cases of a second surgery for HM Group, while in the Control Group, 3 patients required a second surgery for hemostasis. The difference in the rate of secondary surgery between the two groups was not statistically significant (*p* = 0.241, Table 1).
3. Blood loss and irrigation time were compared in HM group with balloon compression(*n* = 4) or with bipolar plasmakinetic hemostasis(*n* = 8), as well as in the control group with balloon compression(*n* = 15). It is evident that patients in either the HM Group or the Control Group who underwent balloon compression had significantly longer postoperative irrigation times compared to patients in the HM Group (*p* = 0.041, Table 2). The average irrigation times for the former two groups were 42.00 ± 12.83 and 46.60 ± 26.73 h, respectively, significantly longer than the average irrigation time of 21.88 ± 3.76 h for patients in the HM Group with bipolar plasmakinetic hemostasis. there was no significant difference between the 3 groups regarding blood loss (*p* = 0.963, Table 2).
4. We further divided the patients into four groups: Hemostasis Management Group with balloon compression(HM group1), Hemostasis Management Group without balloon compression (HM group 2), Control Group with balloon compression (Control group 1), and Control Group without balloon compression(Control group 2). We collected data on absence of median lobe, pre-weight, anticoagulation, surgical time (total surgery duration), postoperative bladder irrigation time, and hospital day after surgery. The details are presented in Table 3.

**Table 1:** Preoperative and postoperative patient information in hemostasis management group (HM group) and control group

	HM group(*n* = 105)	Control group(*n* = 104)	*P* value
Age(year)	72.73 ± 7.90	72.93 ± 7.58	0.553
Psa(ng/ml)	4.66 ± 4.11	5.59 ± 5.82	0.184
pre-weight(g)	61.07 ± 27.44	62.69 ± 28.37	0.674
Sugery time(min)	77.03 ± 29.52	79.07 ± 27.74	0.608
Blood loss(g/L)	16.14 ± 14.54	13.80 ± 9.88	0.175
Post-weight(g)	36.03 ± 18.83	37.03 ± 17.73	0.693
Post-operative hospital day(day)	3.28 ± 0.89	3.43 ± 1.49	0.357
Irrigation time(hour)	21.52 ± 5.36	24.28 ± 13.66	0.0934
postoperative balloon compression (No.)	4	15	0.03*
Second surgery (No.)	0	3	0.241

**Table 2:** The comparison between hemostasis management group with compression, hemostasis management group with bipolar PlasmaKinetic hemostasis and control group

		HM group with compression(*N* = 4)	HM group with bipolar plasmaKinetic hemostasis (*N* = 8)	Control group(*N* = 15)	*P* value
Age(year)	Mean < 75 >=75	75.25 + 2.21 2 2	71.63 + 10.51 7 9	77.40 + 7.49 6 2	0.284
Weight(g)	Mean < 80 g > 80 g	54.25 ± 7.93 4 0	73.25 ± 25.78 5 3	62.13 ± 31.53 10 5	0.500
Blood loss(g/L)	Mean < 15 g/L > 15 g/L	19.25 + 8.30 1 3	17.25 ± 9.75 3 5	18.67 ± 16.31 7 8	0.963
Irrigation time(hour)	Mean < 24 h > 24 h	42.00 ± 12.83 0 4	21.88 ± 3.76 7 1	46.60 ± 26.73 2 13	0.041*

**Table 3:** The comparison between Hemostasis Management Group with balloon compression (HM group1), Hemostasis Management Group without balloon compression (HM group 2), Control Group with balloon compression (Control group 1), and Control Group without balloon compression (Control group 2)

	HM group1	HM group 2	Control group 1	Control group 2
	(n = 4)	(n = 101)	(n = 15)	(n = 89)
Pre-weight (g)	55.50 ± 8.266	61.31 ± 27.92	61.44 ± 30.59	62.92 ± 28.13
Absence of median lobe	0	6	1	6
Anticoagulation	1	26	3	14
Irrigation time (hour)	42 ± 12.83 (18.25*)	20.71 ± 2.75	41.67 ± 16.16	20.52 ± 2.38
Surgery time (min)	86.25 ± 31.98	78.78 ± 27.70	77.07 ± 33.81	76.89 ± 29.01
Blood loss (g/L)	19.25 ± 8.30	16.02 ± 14.75	18.87 ± 16.31	12.98 ± 8.20
Hospital day after surgery (day)	4.25 ± 1.26	3.24 ± 0.86	4.47 ± 3.04	3.26 ± 0.95

## Discussion

While HoLEP offers the advantage of reduced bleeding compared to TURP [3, 9–11], intraoperative bleeding remains a significant concern for surgeons. Choo et al. [12]. found that the most common locations of significant bleeders were at the 3–4 and 8–9 o’clock positions in the proximal prostate and at the bladder neck, particularly at the 3 and 10 o’clock positions. Martin et al. [9]. reported that out of 130 patients who underwent HoLEP surgery, 6 patients required postoperative transfusions, and 4 patients underwent a second operation for hemostasis. Additionally, as the learning curve for HoLEP is steep, many novice surgeons are not able to effectively control intraoperative bleeding [13–15].

Our surgical procedure also encountered bleeding issues, especially due to bleeding from small venous plexuses that cannot be effectively controlled with Holmium laser. In such cases, postoperatively, a urethral catheter balloon compression is often used for hemostasis. Specifically, this involves using a urinary catheter balloon to compress the bleeding area. We inject 30–50 ml of normal saline into the urinary catheter balloon and then pull the catheter and fix it to the patient’s thigh with tape. This procedure results in discomfort and pain on patients. They are also required to lie on bed, which raises the risk of thrombosis [16]. Clearly, this is not an ideal method.

Currently, for patients for whom Holmium laser hemostasis is ineffective during surgery, surgeon resort to use bipolar plasmakinetic technology [7]. However, this is not a routine approach and is not suitable for all situations, not only increases the whole surgery time but also increases the financial burden. Therefore, determining the appropriate timing for intervention with bipolar plasmakinetic becomes crucial. By continuously observing the color of bladder irrigation fluid for 2–3 min, once the criteria mentioned above are met, we immediately apply bipolar plasmakinetic technology. This approach has achieved effective bleeding control. The observation time of 2–3 min is sufficient and should not be too long, as prolonged observation may lead to extensive blood clots covering the surgical field, making it difficult for the surgeon to identify the bleeding area.

In our study, when applying hemostasis procedure during surgery according to the color of irrigation, only 4 of 105 patients received a balloon compression post-operatively, while 15 of 104 patents had to receive a post-operative balloon compression. Although there was no difference in blood loss, the irrigation time of patients received bipolar plasmakinetic hemostasis was significantly lower than those with balloon compression in HM group and control group. In brief, the selective bipolar plasmakinetic hemostasis effectively decrease the post-operative balloon compression and irrigation time. We believe this technique is remarkably beneficial, especially for novice surgeons given the steep learning curve of HoLEP.

One of the limitations of our study is that it was conducted at a single medical center with unparallel assignment of study groups, and it followed a retrospective design rather than a prospective, multi-center approach, which could potentially affect the overall persuasiveness of our research. Furthermore, the sample size of patients in our study was not sufficiently large to enable precise subgroup analyses, such as those based on prostate weight. Meanwhile, a better designed search need be carried out, all pre and post operative criteria should be studied in bivariate and multivariate fashion to study impact of all variables including use of bipolar technology on hemostasis. Thirdly, the assessment of irrigation fluid color was subjectively made by the surgeons, and different surgeons may have varying judgments regarding the color, introducing some degree of variability. Also, accurate calculation of blood loss is a complex process, the calculation in our study may lead to false reading.

## Conclusion

Based on the bladder irrigation color chart, the selective application of bipolar plasmakinetic hemostasis led to a significant reduction in the number of patients requiring postoperative bladder catheter balloon compression. Secondly, the irrigation time of patients who underwent bipolar plasmakinetic hemostasis also decreased.
